# Proteasome inhibition by VR23 enhances autophagic clearance of FUS^P525L^-mediated persistent stress granule in SH-SY5Y cells

**DOI:** 10.1186/s13041-025-01273-z

**Published:** 2026-01-08

**Authors:** Seong Hyun Kim, Jun Hee So, Yong Hwan Kim, Hyo-Sung Kim, Na Yeon Park, Joon Bum Kim, Doo Sin Jo, Eunbyul Yeom, Jin-A Lee, Ji-Eun Bae, Dong-Hyung Cho

**Affiliations:** 1 School of Life Sciences, BK21 FOUR KNU Creative BioResearch Group, Kyungpook National University, 80 Daehakro, Bukgu, Daegu, 41566 Republic of Korea; 2 Organelle Institute, Kyungpook National University, 80 Daehakro, Bukgu, Daegu, 41566 Republic of Korea; 3 Orgasis Corp., Suwon, Gyeonggi-do, Republic of Korea; 4 Department of Biological Sciences and Biotechnology, College of Life Sciences and Nanotechnology, Hannam University, Daejeon, Republic of Korea

**Keywords:** Granulophagy, Stress granules, FUS, VR23, Selective autophagy

## Abstract

Autophagy is a conserved catabolic pathway that preserves cellular homeostasis through lysosomal degradation. Beyond its general role in proteostasis, selective autophagy mediates the clearance of selective cellular targets such as persistent stress granules (SGs), in a process termed granulophagy. SGs are dynamic cytoplasmic assemblies that normally disassemble after stress relief; however, their aberrant persistence has arisen as a pathological feature of neurodegenerative disorders, including amyotrophic lateral sclerosis (ALS). However, the molecular regulation of granulophagy remains incompletely understood. Here, we established a tandem fluorescent SG reporter system with mCherry-pHluorin-FUS^P525L^, enabling live-cell visualization of granulophagic flux. Using this system, we screened a chemical library and identified VR23, a proteasome inhibitor, as a potent inducer of granulophagy. VR23 promoted SG clearance through autophagic mechanisms, as evidenced by enhanced LC3 colocalization, lysosome-dependent degradation, and Bafilomycin A1-sensitive flux. Notably, disruption of SG assembly via G3BP1 inhibition abolished VR23-induced clearance, confirming its SG selectivity. These findings suggest a link between proteasome inhibition and granulophagy, highlighting VR23 as a valuable tool compound to dissect the mechanisms of SG turnover, and provide a platform for discovering modulators of pathological SG clearance in protein aggregation.

**Supplementary Information:**

The online version contains supplementary material available at 10.1186/s13041-025-01273-z.

## Introduction

The maintenance of proteostasis depends on efficient protein quality control systems that eliminate misfolded or unnecessary proteins [1]. When this surveillance fails, aberrant liquid–liquid phase separation (LLPS) and aggregate formation ensue, driving the pathogenesis of a wide range of proteinopathies [2]. Recent studies highlight that LLPS-derived condensates can transition into solid-like aggregates, a phenomenon particularly evident for disease-associated RNA-binding proteins (RBPs) such as FUS and TDP-43 [3, 4]. Among the proteinopathies, amyotrophic lateral sclerosis (ALS) and frontotemporal dementia (FTD) are prototypical neurodegenerative disorders in which RBPs become mislocalized and form toxic assemblies.

One of the most prominent examples is FUS, a nuclear RBP that regulates transcription, RNA splicing, and transport. Under physiological conditions, FUS predominantly resides in the nucleus, but ALS-linked mutations impair its nuclear import and promote cytosolic accumulation [5, 6]. This mislocalization disrupts FUS function and accelerates disease progression [5]. A particularly aggressive variant, FUS^P525L^, causes aberrant cytoplasmic localization, where it aberrantly associates with stress granules (SGs) and forms condensates with increased abundance, altered molecular composition, and enhanced pathological aggregation compared with normal SGs [5–7].

SGs themselves are non-membranous cytoplasmic condensates that assemble transiently in response to diverse cellular stressors. They are primarily composed of untranslated mRNAs, RBPs, and ribosomes [8, 9]. By modulating mRNA translation and localization, SGs basically buffer cells against acute stress and fine-tune signaling pathways to preserve homeostasis [10]. Normally, SGs disassemble after stress removal, but persistent or aberrant SGs interfere with RNA metabolism and protein homeostasis, thereby contributing to proteinopathies associated with ALS or FTD [8]. To counteract cellular stress, cells employ multiple strategies for SG clearance. These include passive dissolution upon stress relief and active degradation through chaperone-mediated dissolution, the ubiquitin–proteasome system as well as autophagy-lysosome pathway [10, 11]. Among these mechanisms, autophagy plays a particularly critical role in targeting persistent SGs, consistent with its conserved function in eliminating misfolded proteins and damaged organelles via lysosomal degradation [12]. Therefore, disruption of this process results in defective SG clearance: depletion of the AAA + ATPase valosin-containing protein (VCP/p97) impairs SG degradation in both yeast and mammalian cells, and ALS-linked VCP mutations (VCP^A232E^, VCP^R155H^) drive constitutive SG accumulation containing eIF3, TDP-43, and VCP itself [13]. Persistent SGs are frequently sequestered into aggresomes for subsequent autophagic degradation [14]. Notably, other autophagy regulators also participate in SG clearance, such as histone deacetylase 6 (HDAC6), which governs SG turnover under viral stress [15], and TRIM21, an E3 ligase that ubiquitinates Ras GTPase-activating protein-binding protein 1 (G3BP1) to suppress SG assembly [16]. Cargo receptors including SQSTM1 and NBR1 further link ubiquitinated SG components to the autophagic machinery, reinforcing SG-selective autophagy (granulophagy) as a dedicated clearance route [13, 14]. Collectively, these findings support on a model in which pathological SGs represent a critical interface between RNA dysregulation and proteostasis collapse in ALS and related neurodegenerative disorders.

Granulophagy serves as a critical quality control process that prevents persistent SGs from transitioning into irreversible aggregates. However, the molecular regulation of granulophagy remains largely undefined. To identify novel regulators for granulophagy, we established a fluorescence-based reporter system and conducted a focused chemical compound library screen. Through this approach, we identified VR23 (C₁₉H₁₆ClN₅O₆S), a known proteasome inhibitor, as a potent inducer of granulophagy that promotes the clearance of FUS^P525L^-associated persistent SGs.

## Materials and methods

### Reagents

Sodium arsenite (Arsenite, S7400) and bortezomib (504314) were obtained from Sigma-Aldrich (St. Louis, MO). FAZ-3532 (HY-162288) was purchased from MedChemExpress (Monmouth Junction, NJ). VR23 was acquired from Selleck chemicals (S7933, Houston, TX, USA). MG132 was supplied by Cayman Chemical (10012628, Ann Arbor, MI, USA). Antibodies against LC3 (1:2¸000, NB100-2220) and ACTIN (ACTA1, 1:10,000, NB60-501) sourced from NOVUS Biologicals (Littleton, CO). G3BP1 antibody was obtained from Proteintech (13057-2-AP, Rosemont, IL, USA). EGFP-FUS^P525L^ plasmid and FLAG-FUS^P525L^ plasmid were generously provided by Dr. Hyung-Jun Kim (Korea Brain Research Institute, Korea) or Dr. Jin-A Lee (Hannam University, Korea). We purchased mCherry-pHluorin plasmid from Addgene (#32001; deposited by Dr, Sergio Grinstein, respectively).

### Cloning and cell lines

The mCherry-pHluorin-FUS^P525L^ construct was synthesized by CosmoGenetech (Seoul, Republic of Korea). The FLAG-FUS^P525L^ fragment was amplified by PCR and subsequently inserted into the mCherry-pHluorin vector using standard molecular cloning Techniques. SH-SY5Y neuroblastoma was obtained from ATCC (Manassas, VA). Cells were cultured at 37 °C in a 5% CO_2_ incubator and maintained in Dulbecco’s Modified Eagle’s Medium (DMEM, WELGENE, Gyeongsan, Republic of Korea) supplemented with 12% fetal bovine serum (FBS, Cytiva, Chicago, IL) and 1% penicillin–streptomycin (WELGENE). For transient expression, SH-SY5Y cells were transfected with EGFP*-*FUS^P525L^ using Lipofectamine 2000 (Invitrogen, Carlsbad, CA, USA) according to the manufacturer’s protocol. To generate stable cell line expressing mCherry-SEpHluorin-FUS^P525L^ in SH-SY5Y cells, cells were transfected with mCherry-pHluorin-FUS^P525L^ using Lipofectamine 2000 (Invitrogen, Carlsbad, CA). Transfected cells were selected with G418 (800 µg/mL) for 7–9 days until resistant colonies appeared. Single-cell clones were then isolated by seeding 1 cell per well in a 96-well plate. After 2 weeks, wells containing uniformly fluorescent colonies were identified under a fluorescence microscope and then expanded. To validate the functionality of the fluorescence reporter system, SG formation was examined under arsenite treatment.

### Cell-based library screening

A ubiquitination-related chemical compound library from TargetMol (L8600, Boston, MA) was used for cell-based chemical library screening. SH-SY5Y/mCherry-pHluorin-FUS^P525L^ cells were seeded in 96-well plates and incubated for 24 h. Each compound from the library was co-treated with arsenite (100 μM) for 1 h. Cells were analyzed under an Operetta CLS high-content imaging system (Revvity, Shelton, CT) at 20 × magnification. SG presence and abundance were evaluated using a five-point grading scale (grade 0–4). Grade 0 indicated no detectable red-SGs (0%), grade 1 indicating minimal activity (< 5%), grade 2 indicated low activity (5–15%), grade 3 reflected moderate activity (15–30%), and grade 4 representing maximal activity (> 30% of cells with red-SGs). During the screening, ~ 50–100 cells per well were evaluated across multiple image fields. Images were acquired using the Operetta CLS system, and the grades were assigned in a semi-manual manner by visual inspection according to the predefined criteria.

### Western blotting

Cell lysates were prepared using 2 × Laemmli sample buffer (#161073, Bio-Rad Laboratories, Hercules, CA). Total protein concentrations were determined using the Bradford assay (#5000006, Bio-Rad). Proteins were separated by 12% SDS-PAGE and transferred onto PVDF membranes. Membranes were incubated overnight with primary antibodies. Horseradish peroxidase (HRP)-conjugated secondary antibodies (Pierce, Rockford, IL; 7074 and 7076, Cell Signaling Technology, Danvers, MA) were used for detection. Chemiluminescent signals were visualized using the Clarity Western ECL substrate (W3680-010, Bio-Rad) and analyzed with CS Analyzer Software 4 (ATTO, Tokyo, Japan).

### Granulophagy evaluation

To validate the granulophagic activity of mCherry-pHluorin-FUS^P525L^ shows granulophagic activity, SH-SY5Y cells were transiently transfected with GFP-tagged FUS^P525L^ and treated with arsenite and VR23 for 1 h. The cells then fixed with 4% paraformaldehyde for 20 min. After washing, cells were blocked with 1% bovine serum albumin (BSA) in PBS and incubated overnight at 4 °C with anti-LC3B antibodies overnight. After washing, cells were incubated with Alexa Fluor 594-conjugated secondary antibodies at room temperature for 1 h. Fluorescence images were captured using a confocal laser scanning microscope (Carl Zeiss, Oberkochen, Germany).

To evaluate granulophagic activity induced by VR23, SH-SY5Y/mCherry-pHluorin-FUS^P525L^ cells were treated with arsenite and VR23 with or without BafA1. Fluorescence images were acquired using confocal laser scanning microscope. The number of red dots and the percentage of cells with red dots were quantified using ImageJ software (NIH, Bethesda, MD, USA).

### Statistical analysis

All experiments were performed at least three times independently. Data are presented as the mean ± SEM. Statistical analyses were conducted using GraphPad Prism 8 (GraphPad Software, San Diego, CA). One-way analysis of variance (ANOVA) was used for statistical comparisons.

## Results

### VR23 promotes granulophagy of FUS^P525L^ in SH-SY5Y cells

Mutations in FUS such as FUS^P525L^ drive its cytoplasmic mislocalization and aberrant phase separations. Recent studies indicate that cytoplasmically mislocalized ALS-linked FUS^P525L^ is robustly recruited to SGs under oxidative stress conditions and induced pluripotent stem cells (iPSC) [5, 6]. To determine whether FUS^P525L^ is incorporated into SGs in SH-SY5Y cells, we transiently expressed EGFP-tagged FUS^P525L^ in SH-SY5Y cells and immunostained G3BP1, a canonical SG marker. Upon arsenite treatment, a standard SG inducer, highly discrete FUS^P525L^ puncta emerged and co-localized with G3BP1, confirming their SG identity (Suppl. Fig. 1A).

**Fig. 1:**
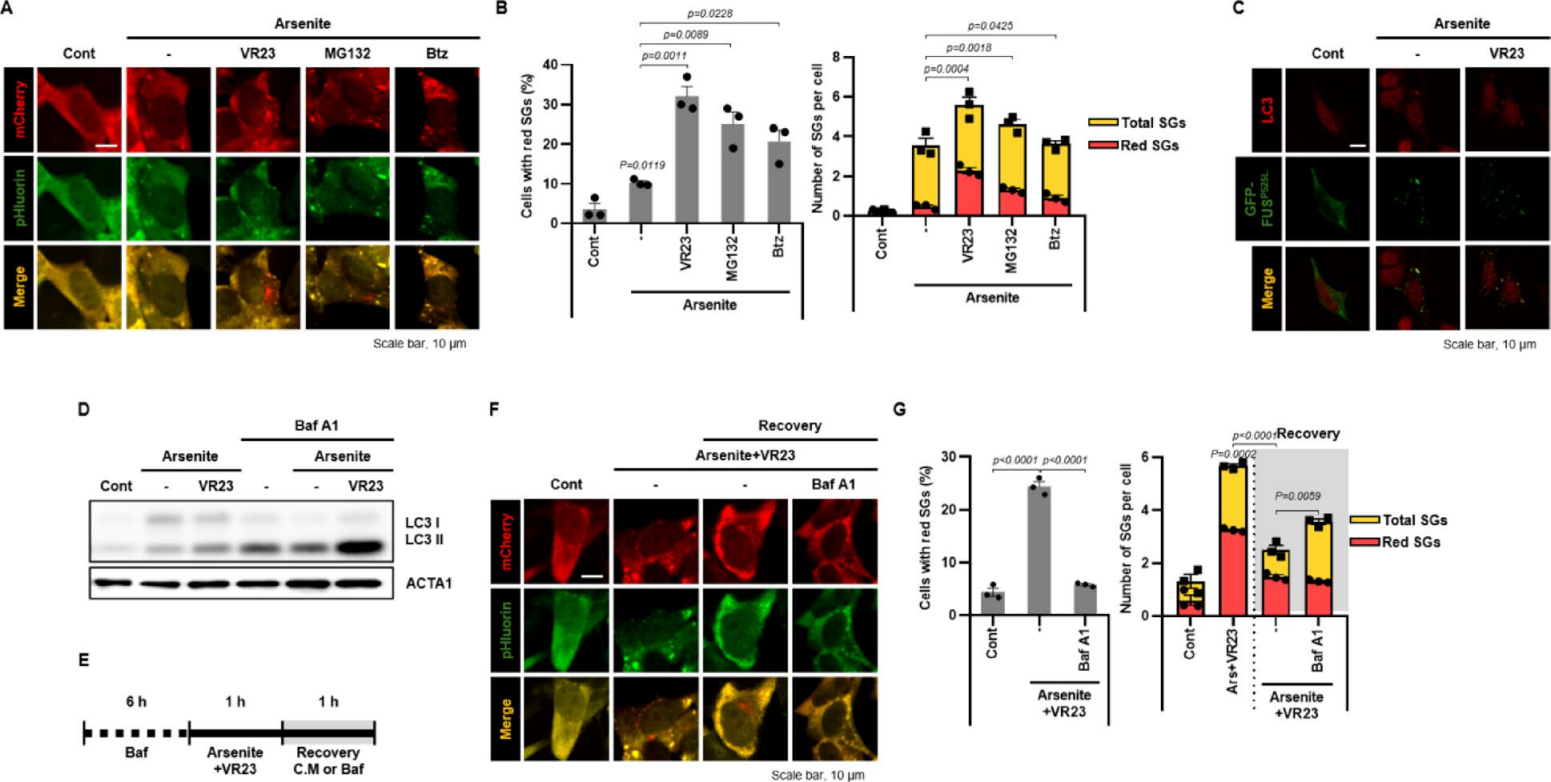
VR23 incresases granulophagy flux in SH-SY5Y/mCherry-pHluorin-FUS^P525L^ cells. (**A, B**) SH-SY5Y/mCherry-pHluorin-FUS^P525L^ cells were treated with arsenite (100 μM) and VR23 (20 μM), MG132 (20 μM), or Bortezomib (Btz, 500 nM) for 1 h. (**A**) Fluorescent cells were imaged to visualize SG localization during the cellular response. Scale bar: 10 μm. **(B**) Quantification of total or red dots per cell and the percentage of cells containing at least one red dot (n ≥ 1) were performed to evaluate SG clearance efficiency under each treatment. (**C**) SH-SY5Y cells transfected with EGFP-FUS^P525L^ were treated with arsenite (100 μM) with or without VR23 (20 μM) for 1 h. Then the cells were immune-stained for LC3 (red). Scale bar: 10 μm. (**D**) SH-SY5Y/mCherry-pHluorin-FUS^P525L^ cells were pre-treated with Bafilomycin A1 (Baf A1, 20 nM) for 6 h. Then, the cells were further exposed to arsenite (100 μM) and VR23 (20 μM). Protein expression levels were analyzed by Western blotting using indicated antibodies. (**E–G**) SH-SY5Y/mCherry-pHluorin-FUS^P525L^ cells were pre-treated with Bafilomycin A1 (Baf A1, 20 nM) for 6 h. And then, the cells were further incubated with arsenite (100 μM) and VR23 (20 μM). **(E**) After 1 h, the cells recovered with complete medium or Bafilomycin A1 (Baf A1, 20 nM) for 1 h. (**F**) Representative images were captured to visualize the cellular response. (**G**) Quantification of total or red dots per cell and the percentage of cells containing at least one red dot (n ≥ 1) were performed to assess the effects of drug treatment on FUS^P525L^ aggregation. Data are presented as mean ± SEM

Then, we developed granulophagy monitoring system using a tandem fluorescence construct, mCherry-pHluorin-FUS^P525L^, and generated a stable cell line in SH-SY5Y (SH-SY5Y/mCherry-pHluorin-FUS^P525L^) cells. This platform exploits the differential pH sensitivity of the fluorophores: pHluorin is acid-labile, whereas mCherry is more acid-stable. During granulophagy, delivery of SGs to acidic lysosomes quenches pHluorin fluorescence but preserves mCherry, thereby enabling real-time visualization of SG degradation. Using this system, we screened a library of ubiquitination-related compounds under conditions of arsenite -induced SG formation. Based on the result, VR23 markedly increased the number of mCherry-only puncta (“red dots”), indicative of enhanced granulophagy (Suppl. Fig. 1B). Since VR23 had not previously been reported to stimulate autophagy, we compared its effects with other proteasome inhibitors, including MG132 and bortezomib in SH-SY5Y/mCherry-pHluorin-FUS^P525L^ cells. Notably, when co-treated with arsenite, all three inhibitors increased the number of red dots and the proportion of red-dot-positive cells relative to arsenite alone; however, VR23 produced the most pronounced effect (Fig. 1A and B). Consistently, co-treatment with arsenite and VR23 significantly increased LC3 and FUS^P525L^ co-localization, supporting VR23-mediated activation of granulophagy (Fig. 1C).

Then, we further investigated granulophagy flux using Bafilomycin A1, an inhibitor of autophagosome-lysosome fusion. As shown in Fig. 1D, LC3-II levels were increased by arsenite and further elevated by co-treatment with VR23. However, Bafilomycin A1 treatment led to a dramatic increase in LC3-II only in the VR23 co-treated group, indicating enhanced autophagic flux in SH-SY5Y cells (Fig. 1D). Finally, we monitored granulophagic flux dynamics using the mCherry-pHluorin-FUS^P525L^ system. After Bafilomycin A1 pretreatment, cells were exposed to arsenite and VR23 for 1 h, followed by recovery in either complete medium or Bafilomycin A1 containing medium (Fig. 1E). Recovery in complete medium led to a reduction in SG number, while Bafilomycin A1 impaired SG degradation, confirming that VR23 promotes granulophagic flux in SH-SY5Y cells (Fig. 1F and G).

### Inhibition of SG formation attenuates granulophagy of FUS^P525L^-mediated persistent stress granules

**Fig. 2:**
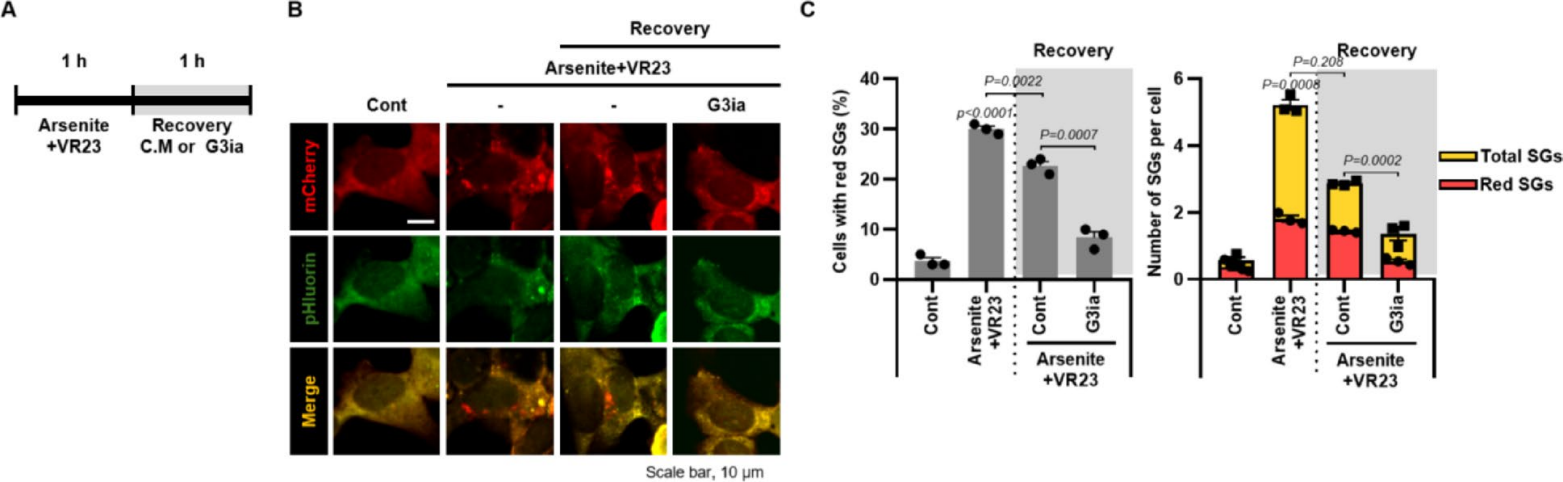
Inhibition of G3BP1 attenuates VR23-mediated granulophagy in SH-SY5Y/mCherry-pHluorin-FUS^P525L^ cells. (**A**–**C**) SH-SY5Y/mCherry-pHluorin-FUS^P525L^ cells were pre-treated with arsenite (100 μM) and VR23 (20 μM). After 1 h, the cells recovered with complete medium or FAZ-3532 (G3Ia, 20 μM) for 1 h. (**B**) Representative images were captured to visualize the cellular response. Scale bar: 10 μm. (**C**) Quantification of total or red dots per cell and the percentage of cells containing at least one red dot (n ≥ 1) were performed to assess the effects of drug treatment on FUS^P525L^ aggregation. Data are presented as mean ± SEM

To determine whether VR23 promotes autophagic degradation of FUS^P525L^-mediated persistent SGs, we employed G3Ia-a small-molecule inhibitor of G3BP1 that blocks SG assembly by binding to the NTF2-like (NTF2L) domain of G3BP1 [17]. As shown in Fig. 2A, SH-SY5Y/mCherry-pHluorin-FUS^P525L^ cells were co-treated with arsenite and VR23 to induce SGs and granulophagic flux, then allowed to recover either in complete medium or in medium supplemented with G3Ia. Notably, G3Ia markedly suppressed SG formation during the recovery phase compared with complete medium (Fig. 2B). Subsequently, quantitative analysis (Fig. 2C) revealed that G3Ia treatment substantially reduced SG burden: both the number of mCherry-only (“red”) SG puncta per cell-our readout of lysosome-delivered SGs and the proportion of red-dot positive cells were substantially decreased relative to recovery in complete medium. Since G3Ia prevents SG assembly upstream, the attenuated red-puncta signal indicates that VR23-driven clearance requires the presence of FUS^P525L^-positive SG substrates rather than causing nonspecific accumulation of mCherry fluorescence. Together, these findings support a model in which VR23 promotes selective granulophagy targeting FUS^P525L^-positive SGs for autophagic degradation.

## Discussion

Granulophagy is increasingly recognized as a crucial cellular safeguard that prevents the persistence of aberrant SGs implicated in neurodegenerative diseases such as ALS [13, 16, 18–20]. However, the molecular mechanisms governing pathological granulophagy, as well as selective regulators capable of triggering granulophagy, remain largely unexplored. Here, we established a tandem-fluorescent SG reporter, mCherry-pHluorin-FUS^P525L^, which enables real-time quantification of granulophagic flux in live cells. Using this platform in a high-content, ubiquitination-focused small-molecule screen, we identified VR23 as a potent inducer of SG clearance (Fig. 1). Compared with conventional LC3-puncta assays, our dual-color reporter system provides higher temporal resolution, distinguishes autophagosome maturation from lysosomal degradation, and is amenable to high-throughput screening [21]. While this approach offers clear advantages, it also depends on proper lysosomal acidification to quench pHluorin, and off-target pH fluctuations or tandem-tag overexpression could distort autophagic readouts [22, 23]. Thus, our findings provide a tractable framework for dissecting granulophagy of pathological SGs.

Mechanistically, VR23 functions as a potent proteasome inhibitor that primarily blocks trypsin-like (β2) activity while also suppressing chymotrypsin- and caspase-like sites, thereby causing intracellular accumulation of ubiquitinated substrates [24]. Such proteasome inhibition induces cyclin E accumulation, centrosome amplification, and apoptotic stress; in our neuronal context, we propose that VR23-induced proteostasis imbalance triggers compensatory activation of selective autophagy to relieve ubiquitin load and eliminate aggregation-prone condensates [24]. Indeed, proteasome inhibition is well-known to engage cross-talk with autophagy pathways, redirecting ubiquitinated substrates toward lysosomal degradation through cargo receptors such as SQSTM1/p62 and NBR1 [16, 19, 25]. Accumulation of polyubiquitinated proteins under VR23 treatment may facilitate their sequestration into FUS^P525L^-positive SGs, thereby marking these condensates for selective autophagic degradation. Consistent with this model, other proteasome inhibitors such as MG132 and bortezomib also elevated granulophagic activity, yet VR23 produced the strongest induction of SG autophagic flux (Fig. 1). These findings indicate that proteasome inhibition promotes lysosome-dependent degradation and that VR23 is particularly effective at promoting granulophagy for FUS^P525L^-mediated SGs.

To date, mechanistic studies on granulophagy have mainly focused on adaptor proteins (VCP/p97, HDAC6, TRIM21) and autophagic cargo receptors (SQSTM1, NBR1), whereas pharmacological modulators remain largely unexplored [13, 15, 16, 19, 26]. Therefore, our identification of VR23 as a chemical enhancer of granulophagy thus provides a proof-of-concept that SG clearance can be modulated pharmacologically. Although the route that links proteasome stress to SG removal is not fully defined, the VR23 response-together with our mCherry-pHluorin-FUS^P525L^ dual fluorescence reporter-provides a practical handle to investigate this connection. Therefore, further work is needed to define how ubiquitin signaling together with cargo receptors (e.g., SQSTM1) orchestrates pathological SG clearance during proteotoxic stress. Advancing this pathway is expected to inform strategies to reduce persistent SGs in proteinopathy-related neurodegeneration.

## Supplementary Information

Below is the link to the electronic supplementary material.

Supplementary Material 1.

## Data Availability

The datasets used and/or analyzed during the current study are available from the corresponding author on reasonable request.

## Abbreviations

SGs: Stress granules
ALS: Amyotrophic lateral sclerosis
LLPS: Liquid-liquid phase separation
RBPs: RNA-binding proteins
FTD: Frontotemporal dementia
FUS: Fused in sarcoma
HDAC6: Histone deacetylase 6
G3BP1: Ras GTPase-activating protein-binding protein 1
