# Supplementary material for: Proteasome inhibition by VR23 enhances autophagic clearance of FUSP525L-mediated persistent stress granule in SH-SY5Y cells
Source: Mol Brain. 2026 Jan 8;19:10. doi: 10.1186/s13041-025-01273-z (PMC12870388; doi:10.1186/s13041-025-01273-z)
Supplement: Supplementary file 1 — Supplementary Material 1. [file 13041_2025_1273_MOESM1_ESM.docx]

***Supplementary Figure***

***
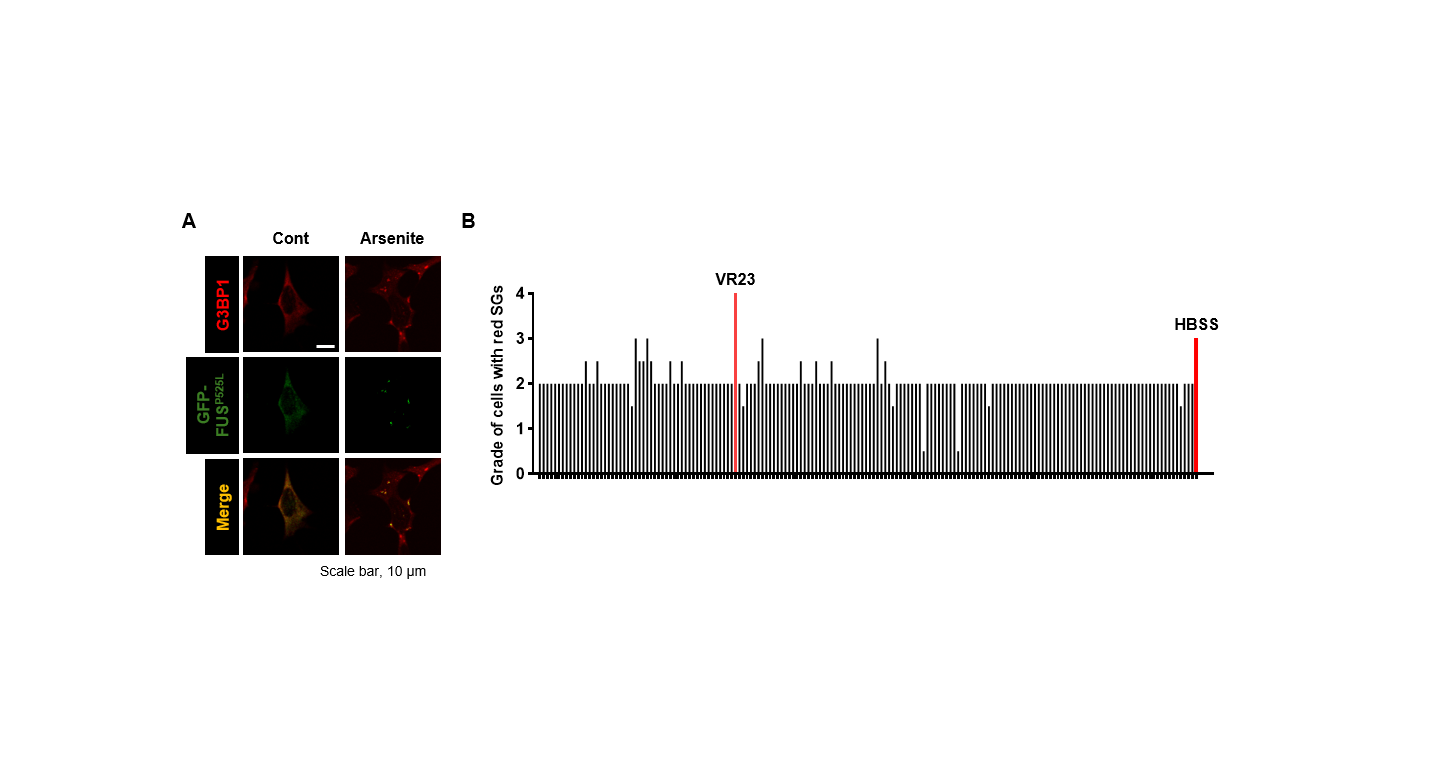
***

***Supplementary Fig. 1. Treatment of sodium arsenite enhances colocalization of G3BP1 with FUS^P525L^ in SH-SY5Y cells***

**(A)** SH-SY5Y cells were transfected with GFP-FUS^P525L^ and then, cells were treated with arsenite (100 μM) for 1 h. Immunofluorescence staining was performed for G3BP1 (red). (**B)** SH-SY5Y/ mCherry-pHluorin-FUS^P525L^ cells were treated with arsenite (100 μM) and a ubiquitination compound library. The extent of SG formation was graded on a scale from 1 to 5, where grade 1 represents minimal activity (less than 10 % of cells with SGs), and grade 5 represents maximal activity (more than 60 % of cells with SGs). G3Ia (20 μM) was used as a positive control for inhibitor of SG formation.
